# In Vitro and In Vivo Wound Healing and Anti-Inflammatory Activities of Babassu Oil (*Attalea speciosa* Mart. Ex Spreng., Arecaceae)

**DOI:** 10.1155/2020/8858291

**Published:** 2020-09-24

**Authors:** José Alex A. Santos, José Wellinton da Silva, Simone Maria dos Santos, Maria de Fátima Rodrigues, Camila Joyce A. Silva, Márcia Vanusa da Silva, Maria Tereza S. Correia, Julianna F. C. Albuquerque, Cristiane M. L. Melo, Teresinha G. Silva, René D. Martins, Francisco Carlos A. Aguiar Júnior, Rafael M. Ximenes

**Affiliations:** ^1^Departamento de Enfermagem, Instituto Federal de Pernambuco, Abreu e Lima 53.515-120, Brazil; ^2^Departamento de Antibióticos, Universidade Federal de Pernambuco, Recife 50.740-525, Brazil; ^3^Departamento de Bioquímica, Universidade Federal de Pernambuco, Recife 50.670-901, Brazil; ^4^Centro Acadêmico de Vitória, Universidade Federal de Pernambuco, Vitória de Santo Antão 55.608-680, Brazil

## Abstract

Babassu (*Attalea speciosa* Mart. ex Spreng., Arecaceae) is a palm tree endemic to Brazil and found mainly in the borders of Amazon forest, where the harvesting of its fruits is an important source of income for more than 300,000 people. Among the communities of coconut breakers women, babassu oil is used in culinary, as fuel, and mostly as medicinal oil for the treatment of skin wounds and inflammation. This study aimed to evaluate in vitro and in vivo the wound healing effects of babassu oil. In vitro, babassu oil increased the migration of L929 fibroblasts, inhibited the production of nitric oxide by LPS-stimulated peritoneal macrophages, and increased the levels of INF-*γ* and IL-6 cytokines production. In vivo, babassu oil accelerated the healing process in a full-thickness splinted wound model, by an increase in the fibroblasts number, blood vessels, and collagen deposition in the wounds. The babassu oil also increased the recruitment of inflammatory cells into the wound site and showed an anti-inflammatory effect in a chronic ear edema model, reducing ear thickness, epidermal hyperplasia, and myeloperoxidase activity. Thus, these data corroborate the use of babassu oil in folk medicine as a remedy to treat skin wounds.

## 1. Introduction

Babassu is a palm tree endemic from Brazil. Its oil is used in the manufacturing of food, cosmetics, and pharmaceuticals, being the main product from extractive sources used in the industry in the world [1]. The harvesting of babassu fruits is a predominantly women activity, which are known as “coconut breakers women”. It is the primary source of income for more than 300,000 people in Northeast Brazil, mainly in the Maranhão State, in the borders of the Amazon rain forest [2] and also in other more isolated areas, as in the Araripe Region [3].

Like the oils of other Arecaceae fruits, babassu oil is a rich source of medium-chain saturated fatty acids, especially lauric acid. Among the communities of coconut breakers women, babassu oil is used in culinary, but mostly as medicinal oil, which is used in the treatment of skin wounds and joint and muscular inflammation, among others [2, 4, 5]. Many authors have been reporting the pharmaceutical as the employment as an excipient in different pharmaceutical formulations, as microemulsion [6, 7], and biological properties, as the antimicrobial [8, 9], anti-inflammatory [7, 10], and emollient effects of babassu oil [11].

The healing of skin wounds is a well-orchestrated process usually divided into three partially overlapping stages: inflammatory, proliferative, and remodeling phases. The wound healing success lies in maintaining the wound sterility as well as limiting the oxidative stress and controlling the moisture of the wound to aloud the recruitment of fibroblasts and collagen synthesis [12]. Immune cells help this process through cytokines and chemokines secretion and the phagocytosis of foreign bodies and microorganisms [13].

Despite the widespread use of medicinal plants and their derived products in the treatment of skin wounds in folk medicine and the recognition that phytochemicals can have positive effects on wound healing [14], the effect of babassu oil on skin wounds has not been scientifically evaluated until now. Thus, this study aimed investigates in vitro and in vivo the wound healing effects of babassu oil as well as evaluate its anti-inflammatory activity on chronic skin lesions.

## 2. Materials and Methods

### 2.1. Plant Material and Oil Extraction

Babassu (*Attalea speciosa* Mart. ex Spreng., Arecaceae) fruits were collected by the botanist Alexandre Gomes da Silva in the Catimbau National Park, Buíque, Pernambuco (08° 37′ 23″ S; 37° 09′ 21″ O) in March 2016 (SISBIO authorization no. 26,743-3). A voucher specimen was identified by O. Cano and deposited in the Herbarium Dárdano de Andrade Lima (IPA no. 90,472). Governmental authorization to access the traditional knowledge associated to the uses of babassu was also obtained (SISGEN no. A&D&D31). Babassu coconuts were broken using a hatchet (Tramontina®A&Brazil) to remove the kernels, which were dried in a forced-air oven at 40ºC for 24 h.

The babassu oil was extracted from the dried kernels (250 g) using a benchtop oil expeller (Piteba®, the Netherlands), without heating. To remove solid impurities, the oil was centrifuged at 2,000 g for 10 min, yielding 34%. For all in vitro assays, babassu oil was weighted and a stock solution was prepared in DMSO (maximal final concentration of 0.5%).

### 2.2. Physicochemical Characterization and Fatty Acid Profile

Physicochemical characterization of babassu oil was determined according to the Instituto Adolf Lutz [15] as follows: relative density was measured using a 10 mL pycnometer at 25°C; refraction index was determined using a Abbé refractometer at 40°C; acid values were determined by titration with KOH 0.1 M; peroxide values were calculated from the iodine release from potassium iodide; while the lipid oxidation (rancidity) was determined by Kreis reaction using phloroglucinol in acid medium.

To determine the fatty acid profile of the babassu oil, 25 mg samples were transesterified using 0.5 mL of KOH 0.5 M in methanol. Then, fatty acid methyl esters (FAMEs) were extracted with *n*-hexane.

The samples were analyzed using an Agilent Technologies 7890 Gas Chromatograph coupled with a Flame Ionization Detector (GC-FID) (Palo Alto, CA, USA) equipped with a nonpolar DB-5 ms column (30 m length × 250 *μ*m diameter × 0.25 *μ*m). The oven was initially held at 150°C for 4 min, increased to 280°C by 4 °C/min. The final temperature was maintained for 5 min. The carrier gas was helium supplied at a constant flow of 1 mL/min, and 1 *µ*L sample was automatically injected in split mode (100 : 1) with the injector maintained at 300°C. A standard fatty acid methyl ester mixture (Supelco®, 37 Component FAME Mix, Bellefonte, PA, USA) was used to identify the fatty acid methyl esters.

### 2.3. Calcium and Zinc Levels

Inductively coupled plasma optical emission spectrometry (7000 ICP-OES, ThermoFisher Scientific, Waltham, MA, USA) was used to quantify the levels of calcium and zinc in the babassu oil as described by the Environmental Protection Agency of the United States Method 200.7 [16].

### 2.4. Animals

Male Swiss mice (*n* = 38), 8 weeks old, weighing 3035 g, and male Wistar rats (*n* = 60), 8 week-old, weighing 200300 g, both obtained from the Animal Facilities of UFPE, were used. The animals were maintained in environment-controlled rooms at 24 ± 2ºC, about 55% relative air humidity, 12 h light/dark cycle, with food and water *ad libitum*. All experimental protocols were in accordance with Brazilian laws and were previously approved by the Ethics Committee on Animal Use (CEUA/UFPE, protocol no. 23076.003137/2016-11).

### 2.5. In Vitro Wound Healing Activity

#### 2.5.1. Cell Line and Culture

Mouse L929 fibroblast cell line was obtained from the Cell Bank of Rio de Janeiro (BCRJ) and cultured in Dulbecco's Modified Eagle Medium (DMEM), supplemented with 10% fetal bovine serum (FBS), 2 mM glutamine, 100 U/mL penicillin, and 100 *μ*g/mL streptomycin and maintained at 37°C in humidified atmosphere with 5% CO_2_.

#### 2.5.2. Cell Viability

The effect of babassu oil on L929 cell viability was determined by the method of 3-(4, 5-dimethyl-2-thiazolyl)-2, 5-diphenyl-2H-tetrazolium bromide (MTT). Firstly, L929 cells (3 × 10^5^ cells/mL) were plated in 96-well tissue culture plates for 24 h. Then, babassu oil (100–1.56 *µ*g/mL) was added to the wells, and the cells were incubated for 24 h, 48 h, and 72 h. DMSO 0.5% was used as control. After each interval, 25 *µ*L of MTT solution (5 mg/mL) was added to the wells, and the plates were incubated for another 3 h. At the end of that period, the supernatant was aspirated and 100 *μ*L of DMSO was added in each well for the dissolution of the formazan crystals. The absorbance was measured at 560 nm in a microplate reader. Babassu oil was tested in triplicate in three independent experiments.

#### 2.5.3. Scratch Assay

The scratch assay was used to evaluate the effect of babassu oil on the migration of L929 fibroblasts. Briefly, L929 cells (2 × 10^4^ cells/mL) were plated in 24-well tissue culture plates for 24 h to reach confluence. Cell monolayers were scratched using a sterile 200 *µ*L pipette tip to produce a wounded area with 1,2001,500 *µ*m width. Then, the wells were washed with fresh medium without FBS to remove any unattached cells. Babassu oil (1.56, 3.12, and 6.25 *µ*g/mL) and 10% FBS, used as positive control, were added to the wells in triplicate. Cell migration was measured each 6 h using an inverted microscope Novel XD202 under 40x magnification [17]. The scratch closure was calculated as individual areas under the curve (AUC_24h_) [18].

#### 2.5.4. Isolation of Thioglycolate-Elicited Murine Peritoneal Macrophages

Mice were injected with sterile 3.8% thioglycolate medium (i.p.), and after 72 h, the macrophages were collected from the peritoneum using 10 mL of cold PBS (pH 7.4). The cells were washed twice with PBS and then resuspended in DMEM supplemented with 10% FBS and antibiotics [19]. The effects of babassu oil on cell viability were assessed as described above.

#### 2.5.5. Nitric Oxide and Cytokine Production by LPS-Stimulated Macrophages

The isolated macrophages (3 × 10^5^ cells/mL) were plated in 96-well tissue culture plates for 24 h. After this period, the cells were stimulated with 5 *µ*g/mL lipopolysaccharide from *E. coli* 055:B5 for 1 h and then treated with babassu oil (3.12, 6.25, and 12.5 *µ*g/mL), dexamethasone (10 *µ*g/mL), or *N*_*ω*_-nitro-L-arginine methyl ester (L-NAME, 25 *µ*g/mL). After 24 h, the medium was removed and used to measure the levels of nitrite by the Griess reaction. Th1, Th2, and Th17 cytokines were also quantified in the medium using a BD™ Cytometric Bead Array (BD Biosciences, CA, USA).

### 2.6. In Vivo Wound Healing Assay

#### 2.6.1. Experimental Groups

For the evaluation of babassu oil wound healing effect, rats were randomly allocated into five groups (*n* = 12): group I received 1% Tween 80; group II received Dersani® (commercial oily lotion containing medium-chain triglycerides, sunflower oil, lecithin, retinol, and tocopherol, as positive control); groups III-V received babassu oil (10, 30, and 100%). The animals were treated topically once a day, with 100 *µ*L until the 3^rd^ day.

#### 2.6.2. Splinting Full-Thickness Wound Model

To assess the wound healing activity of babassu oil, a splinting full-thickness wound model in rats was used. The animals were anesthetized with xylazine (10 mg/kg, i.p.) and ketamine (100 mg/kg, i.p.) and had the dorsal hair removed using a clipper followed by a depilatory cream. Two full-thickness wounds were made in each rat using a 6 mm diameter biopsy punch, and then two silicone rings were sutured around the wounds to prevent wound contraction [20]. After recovery from anesthesia, animals were individually caged and observed for 14 days. At the 3^rd^, 7^th^, and 14^th^ days, four rats from each group were euthanized, and the wound was removed for histological analysis.

The clinical evaluation of the wounds was made daily using semiquantitative scores (0 for absent; 1 for discrete; 2 for moderate; and 3 for intense), considering the presence of edema, erythema, scab, and re-epithelization. As the silicone ring prevented the wound contraction, the wound area was not measured.

### 2.7. In Vivo Topical Anti-Inflammatory Activity

Ear edema induced by multiple applications of croton oil was used to assess the topical anti-inflammatory of babassu oil. Shortly, mice received 20 *µ*L of a 5% croton oil in acetone on both ears on alternate days for 9 days. From day 5, mice were treated topically with 10 *µ*L/ear of acetone or babassu oil twice a day. Positive control received 0.1 mg/ear of dexamethasone dissolved in acetone. On the 9^th^ day, 6 h after the administration of croton oil, mice were euthanized, and 6 mm diameter samples were collected from each ear using a biopsy punch. Both samples were weighed for the determination of edema.

The left ear samples were processed for histological and histomorphometric evaluation, while the right ear samples were homogenized in 50 mM phosphate buffer containing 0.5% hexadecyltrimethylammonium bromide (HTAB) for the quantification of myeloperoxidase activity [21].

### 2.8. Histological Analyses

Wounds and ear samples were fixed in 10%-buffered formalin overnight, dehydrated in increasing concentrations of ethanol, diaphanized in xylene, embedded in paraffin, and cut into 5 *µ*m slides, which were stained with hematoxylin and eosin (HE) or Masson's trichrome (MT) and analyzed using an Axiostar Plus optical microscope (Leica, Germany). For the histomorphometric analysis, twenty microphotographs were taken per slide. For wound samples, inflammatory cells (neutrophils, lymphocytes, and macrophages), fibroblasts, and blood vessels were quantified in HE-stained slides, while collagen (%) was quantified in MT-stained slides. For ear samples, dermis and epidermis thickness were measured. Both analyses were performed using ImageJ v1.52 (NIH, MD, USA) with the plugins “cell counter” and “cell deconvolution”.

### 2.9. Statistical Analyses

The data were expressed as mean ± SD and analyzed by ANOVA followed by Bonferroni post-test or by KruskalWallis test, both considering significant values of ^*∗*^*p* < 0.05. All statistical analyses were performed using the software Prism 7.0 (GraphPad, San Diego, CA, USA).

## 3. Results and Discussion

### 3.1. Chemical Characterization

The use of medicinal plants and their derivatives is increasing, and approximately one-third of all traditional herbal medicines are intended for wound treatment [22]. Due to the facility to obtain and the desirable physicochemical properties, oils are one of the most common medicinal preparations used in traditional medicine around the world. Medicinal oils may be from vegetal, animal, and mineral sources or evenly herbal oily extracts made from different plant organs [23]. As a significant part of the vegetable oils, babassu oil is extracted from the dried kernels by cold pressing, and due to its high lauric acid content, it is very stable to oxidation, as evidenced by its peroxide and rancidity values (Table 1). Besides its physicochemical properties, the fatty acid profile of the babassu oil was also determined, and it was evident that there were not marked composition differences with the data reported in the literature, lauric, oleic, and myristic acids being the major compounds found (Table 2), as described by De Oliveira et al. [24]. Zinc and calcium levels, which play an important role in wound healing, were determined by ICP-OES at < 4.85 and 18.0, respectively. Both minerals are found in babassu kernels at levels up to 28.5 mg/kg for zinc [24] and 457.5 mg/kg for calcium [25]. The relative low concentrations of both divalent cations in the oil confirm the low amount of free fatty acids, as shown in Table 1.

### 3.2. In Vitro Wound Healing and Anti-Inflammatory Activity

Despite some criticism on using in vitro assays as single models to study wound healing, a variety of relevant tests can be combined as a first screening to avoid the unnecessary use of laboratory animals. The healing of wounds involves many processes such as inflammation, cell proliferation, matrix deposition, and necessity of limiting oxidative stress and the proliferation of pathogens [26]. Here, it was decided to evaluate the effects of babassu oil on fibroblast migration and macrophage production of nitric oxide and cytokines and its radical scavenging and antimicrobials activities. Both antioxidant and antimicrobial activities were not found in relevant concentrations (data not shown), differently from those described by Nobre et al. [9], where solvent-extracted babassu oil showed MIC values of 32 and 512 *µ*g/mL for different drug-resistant *Staphylococcus aureus* and *Escherichia coli* isolates from surgical wounds. The difference in the antibacterial activity of these two babassu oils is probably due to the fatty acids with ten or fewer carbon atoms, such as caproic (C6:0), caprylic (C8:0), and capric (C10 : 0) acids. These fatty acids correspond to 20.3% of the solvent-extracted oil described by Nobre et al. [9] and only 9.9% of the cold-pressed oil used in this study (Table 2).

**Figure 1:**
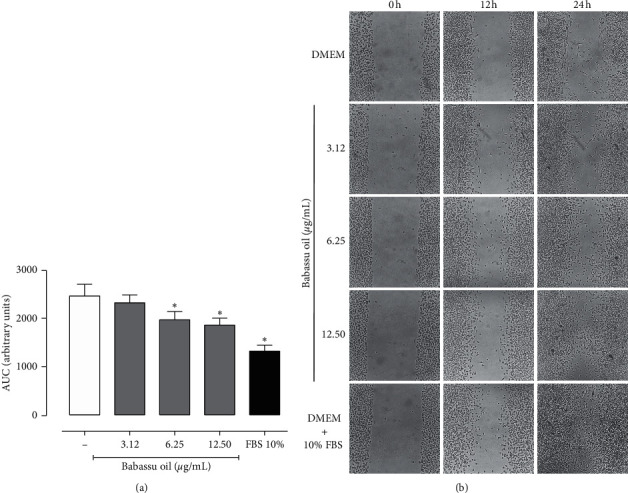
Effect of babassu oil on the migration of L929 fibroblasts in the scratch assay. (a). Area under the curve (AUC) expressed as mean ± SD and analyzed by ANOVA with Bonferroni post hoc. ^*∗*^*p* < 0.05. (b). Representative photomicrography of the different experimental groups at 0, 12, and 24 h.

In vitro, babassu oil was not cytotoxic in concentrations up to 100 *µ*g/mL for both L929 fibroblasts and murine peritoneal macrophages (MPMs). However, concentrations above 25 *µ*g/mL cause an increase in MTT metabolism in L929 cells, which could indicate cell proliferation. This effect was not observed in MPM, which may be beneficial to prevent switching to unfavorable phenotypes, delaying the wound healing process [27]. In the scratch assay, babassu oil increased the cell migration at 6.25 and 12.5 *µ*g/mL, as shown by the smaller areas under the curve (AUC) in Figure 1(a) and by the representative microphotographs shown in Figure 1(b). Ibrahim et al. [28] showed a similar effect of fermented virgin coconut oil (FVCO) in human normal colonic fibroblasts (CCD-18) at concentrations between 3.12 and 25 *µ*g/mL, without any signals of cytotoxicity. In addition, the authors showed that at 6.25 and 12.5 µg/mL, FVCO also increased the formation of new blood vessels in an ex vivo model. Guidoni et al. [29] evaluated the wound healing potential of a vegetable oil blend of flaxseed oil (15%), blackcurrant oil (10%), olive oil (20%), rosehip oil (10%), macadamia oil (15%), and sunflower oil (30%). In the scratch, this oil blend increased fibroblast migration in a concentration-depended manner up to 200 *µ*g/mL.

Since inflammation plays an essential role in wound healing and chronification, LPS-stimulated peritoneal macrophages were used to evaluate the anti-inflammatory activity of babassu oil in vitro (Table 3). Babassu oil inhibited the production of NO by macrophages, which could be beneficial in the healing process since NO is an important chemoattractant during the initial inflammatory phase, with iNOS expression peaking until 48h of wounding [30]. Increased NO level induces keratinocyte apoptosis, while the proper modulation of NO is crucial for the postwounding angiogenesis [31]. Deakin et al. [32] showed that NO inhibition increases IL-6 levels in LPS-stimulated macrophages. IL-6 plays a vital role in all wound healing phases, including proliferation and remodeling phases, and not only in the inflammatory phase [33]. However, an excess of IL-6 signaling may lead to keloid scarring [34]. Babassu oil also increased the levels of IFN-*γ* and TNF-*α*, both known for enhancing wound healing. Treatment with IFN-*γ* results in faster restoration of tissue integrity in both full-thickness incision skin wound models [35, 36]. TNF-*α* plays a dual effect in wound macrophages; in the early phase, it induces M1-phenotype through NF-k*B* signaling to promote host defense, while in the later phase, it induces M2-phenotype through INF-*γ* signaling to promote resolution and healing [37]. Membrane-bound TNF-*α* is responsible for neutrophil apoptosis induced by wound macrophages, decreasing the oxidative stress in the wound bed [38]. Another cytokine modulated by babassu oil was IL-2, which reduces inflammation in the wounds and prevents over deposition of extracellular matrix, preventing aberrant wounds [39]. This result set incited us to further evaluate the in vivo wound healing effects of babassu oil.

### 3.3. In Vivo Wound Healing and Anti-Inflammatory Activity

Unlike human mechanism, which close full-thickness skin wound by scar formation, mice and rats heal primarily by contraction of panniculus carnosus muscle. Thus, to avoid misinterpretation of the effects of babassu oil on wound healing, a full-thickness splinted wound model was used to prevent wound contraction and allowed the wound to heal by granulation and re-epithelialization, similar to what happens in humans [20].

**Figure 2:**
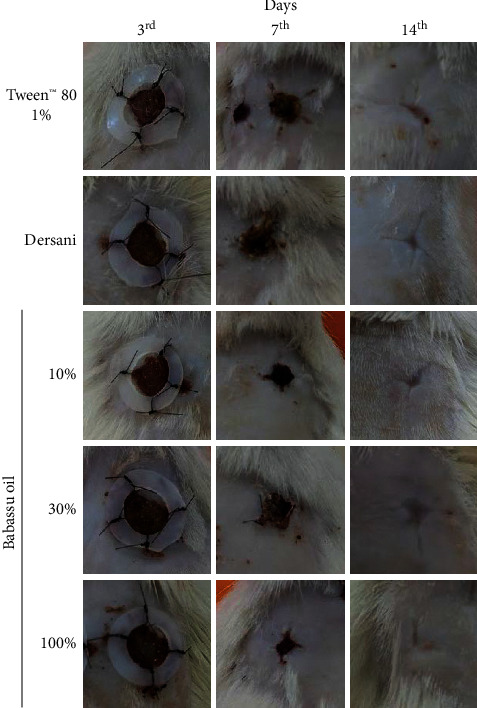
Macroscopic parameters of the wound treated with babassu oil (*Attalea speciosa* Mart. ex Spreng, Arecaceae).

**Figure 3:**
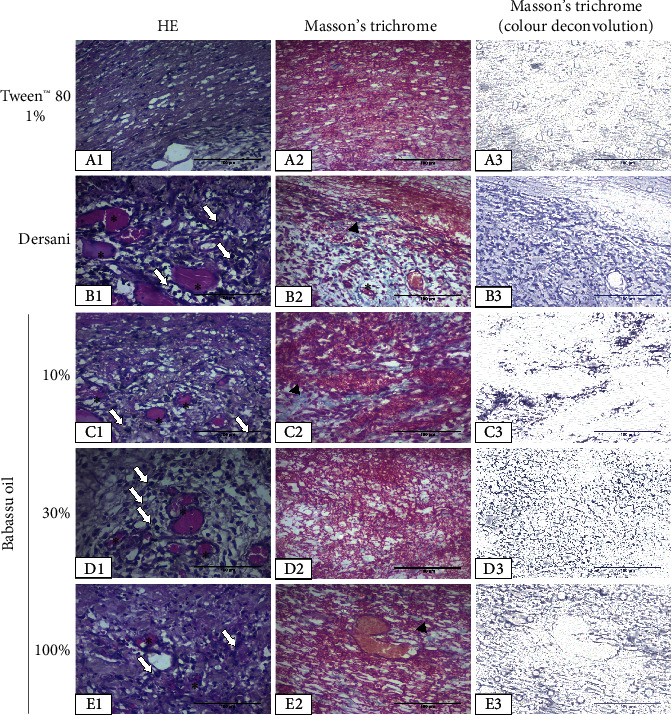
Representative photomicrographs (400x) of the wounds on the 3^rd^ day of treatment with babassu oil (*Attalea speciosa* Mart. ex Spreng, Arecaceae). *White arrows* indicate inflammatory cells; *arrowheads* indicate collagen fibers; and the asterisk indicates blood vessels.

Macroscopically, babassu oil decreased the erythema on the third day and the scab formation in the 14^th^ day after wounding. At the same time, it increased the reepithelization at the end of the experiment (Figure 2 and Table 4). In the histomorphometric analysis, babassu oil increased neutrophil infiltration on the 3^rd^ day, decreasing the mononuclear infiltration after 7 and 14 days. It stimulated the blood vessels formation at all periods analyzed and increased the count of fibroblasts and collagen deposition after 7 and 14 days (Figure 3 and Table 5).

Nevin and Rajamohan [40] showed that coconut oil was able to accelerate wound healing in rats, increasing the collagen deposition and limiting oxidative stress in the wound tissue. The authors attributed the effect to the presence of medium-chain fatty acids (C:6 to C:12) and also to minor compounds found in the nonsaponifiable fraction of the oil, such as polyphenols, vitamin E, and provitamin A. Both major fatty acids found in babassu oil and lauric and oleic acids have been reported to positively affect wound healing. Lauric acid and its monoglyceride are known for their antimicrobial properties, which could maintain the wound bed's necessary sterility for the healing process [41]. Oleic acid has also been reported to modulate the immune response in wound healing through upregulation of collagen, matrix metalloproteinase-9 (MMP-9), IL-10, and TNF-*α* and downregulation of cycloxygenase-2 (COX-2) expressions, in addition to a decrease in the inflammatory infiltrate after 5 days [42]. On the contrary, Pereira et al. [43] described a proinflammatory effect of oleic acid topical administration on rat skin wounds on the first days, which may speed up the healing process. This result agrees with the increase in inflammatory cells in the wounds treated with babassu oil and Dersani (Table 5).

**Figure 4:**
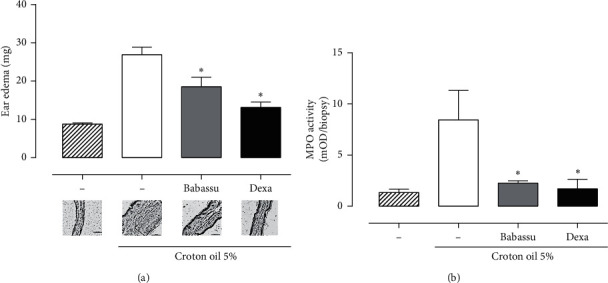
Chronic ear edema induced by multiple applications of croton oil in mice. (a). Ear edema and representative micrographs. (b). Myeloperoxidase (MPO) activity in ear samples. Data were analyzed by ANOVA followed by Bonferroni posttest, considering ^*∗*^*p* < 0.05.

To better understand the role of babassu oil on skin inflammation, a chronic croton oil-induced ear edema was performed. In this model, babassu oil decreased the edema and the myeloperoxidase activity on the 9^th^ day of the experiment (5^th^ day of treatment) (Figure 4). The histomorphometric analysis showed that the ears treated with babassu oil or dexamethasone were narrow than those who received only acetone. The same effect was observed in the epidermal thickness (Table 6). Reis et al. have already shown the acute anti-inflammatory effects of babassu oil and lauric acid on mouse ear edema models, attributing those effects to inhibition of arachidonic acid metabolism and histamine/serotonin release.

## 4. Conclusions

In conclusion, babassu oil stimulates L929 fibroblasts migration and modulates the inflammatory response induced by LPS in mice peritoneal macrophages. *In vivo*, babassu oil was able to accelerate the healing process in a full-thickness splinted wound model due to an increase in the fibroblasts number, blood vessels, and collagen deposition in the wounds. The babassu oil also increased the recruitment of inflammatory cells into the wound site and showed an anti-inflammatory effect in a chronic ear edema model, reducing the ear thickness, epidermal hyperplasia, and myeloperoxidase activity. Thus, these data corroborate the use of babassu oil in folk medicine as a remedy to treat skin wounds.

## Figures and Tables

**Table 1:** Physicochemical parameters of babassu oil (*Attalea speciosa* Mart. ex Spreng, Arecaceae) from Catimbau National Park, Brazil.

Physicochemical parameters	Babassu oil (unrefined)	Reference values (refined oil)
Relative density (g/mL)	0.9280	0.9140–0.9170
Refractive index at 40°C	1.451	1.448–1.451
Acid value (mgKOH/g)	0.14	Max. 4
Peroxide value (meq/kg)	nd	Max. 15
Rancidity	Absent	Absent

nd: not detected.

**Table 2:** Fatty acid profile of the babassu oil (*Attalea speciosa* Mart. ex Spreng, Arecaceae) from Catimbau National Park, Brazil.

Skeleton	Compound	Area (%) ± SD
C8:0	Caprylic acid	4.59 ± 0.29
C10 : 0	Capric acid	5.33 ± 0.14
C12 : 0	Lauric acid	46.05 ± 0.61
C14 : 0	Myristic acid	15.04 ± 0.04
C16 : 0	Palmitic acid	8.26 ± 0.17
C18 : 1n9c	Oleic acid	15.22 ± 0.39
C18 : 2n6c	Linoleic acid	2.71 ± 0.48
C18 : 0	Stearic acid	2.80 ± 0.06

**Table 3:** Nitric oxide (NO) and cytokines released by LPS-stimulated macrophages.

	(*µ*g/mL)	NO (*µ*M)	INF-*γ* (pg/mL)	TNF-*α* (pg/mL)	IL-2 (pg/mL)	IL-6 (pg/mL)
PBS	—	0.03 ± 0.01	<0.5	<0.9	496.0 ± 110.4	<1.4
LPS	—	1.15 ± 0.19	980.4 ± 38.6	<0.9	516.4 ± 105.7	584.4 ± 18.4
Babassu oil	3.12	0.31 ± 0.12^*∗*^	2200.1 ± 82.8^*∗*^	107.7 ± 19.2^*∗*^	245.0 ± 133.9	1286.1 ± 25.9^*∗*^
	6.25	0.28 ± 0.10^*∗*^	2214.2 ± 28.5^*∗*^	<0.9	310.5 ± 90.6	1266.3 ± 8.6^*∗*^
	12.5	0.29 ± 0.09^*∗*^	1925.6 ± 7.1^*∗*^	26.3 ± 45.6	578.9 ± 110.9	1197.4 ± 11.8^*∗*^
DEXA	10	0.72 ± 0.12^*∗*^	<0.5^*∗*^	<0.9	619.2 ± 157.0	7.3 ± 1.3^*∗*^
L-NAME	25	0.31 ± 0.05^*∗*^	—	—	—	—

Data were analyzed by ANOVA followed by the Bonferroni posttest, considering significant values of ^*∗*^*p* < 0.05 when compared with group LPS. —: not tested.

**Table 4:** Macroscopic parameters of the wound treated with babassu oil (*Attalea speciosa* Mart. ex Spreng, Arecaceae).

Parameters	Period (days)	Groups-Median (min-max)				
		Tween™ 80	Dersani	Babassu oil		
		1%		10%	30%	100%
Edema	3^rd^	1 (1-2)	2 (1–3)^*∗*^	2 (1–2)^*∗*^	2 (1–2)^*∗*^	2 (1–3)^*∗*^
	7^th^	1 (0-2)	1 (0–1)	1 (0–2)	1 (0–1)	1 (0–2)
	14^th^	0 (0-0)	0 (0-0)	0 (0-0)	0 (0-0)	0 (0–0)

Erythema	3^rd^	1 (1-2)	2 (1–2)^*∗*^	2 (1–2)^*∗*^	1 (1–2)	1 (1–2)
	7^th^	0 (0-1)	1 (0–1)^*∗*^	0 (0–1)	0 (0–1)	1 (0–1)
	14^th^	0 (0-1)	0 (0–1)	0 (0–0)^*∗*^	0 (0–0)^*∗*^	0 (0-1)

Scab	3^rd^	3 (2-3)	3 (2–3)	2 (2–3)	3 (2–3)	3 (2–3)
	7^th^	3 (3-3)	3 (3–3)	3 (3–3)	3 (3–3)	3 (3–3)
	14^th^	1 (0-1)	0 (0–1)^*∗*^	0 (0–0)^*∗*^	0 (0–0)^*∗*^	0 (0–0)^*∗*^

Epithelialization	3^rd^	0 (0-0)	0 (0–0)	0 (0–0)	0 (0–0)	0 (0–0)
	7^th^	2 (1-2)	2 (1–2)	2 (1–2)	2 (1–2)	2 (1–2)
	14^th^	2 (2-3)	3 (2–3)^*∗*^	3 (2–3)^*∗*^	3 (2–3)^*∗*^	3 (2–3)^*∗*^

Data were analyzed by the MannWhitney test, considering significant values of ^*∗*^*p* < 0.05 when compared with group Tween™ 80 1%.

**Table 5:** Histomorphometric parameters of wounds treated with babassu oil (*Attalea speciosa* Mart. ex Spreng, Arecaceae).

Parameters	Period (days)	Groups–(mean ± SEM)				
		Tween™ 80	Dersani	Babassu oil		
		1%		10%	30%	100%
Inflammatory cells (no./micrograph)	3^rd^	121.8 ± 1.2	138.3 ± 2.6^*∗*^	128.5 ± 1.5^*∗*^	130.9 ± 1.7^*∗*^	130.6 ± 1.8^*∗*^
	7^th^	66.7 ± 1.0	65.9 ± 0.6	62.0 ± 0.4^*∗*^	61.1 ± 0.3^*∗*^	63.5 ± 0.4^*∗*^
	14^th^	29.8 ± 0.1	33.6 ± 0.4^*∗*^	31.5 ± 0.2^*∗*^	31.2 ± 0.2^*∗*^	33.8 ± 0.3^*∗*^

Blood vessels (no./micrograph)	3^rd^	11.1 ± 0.3	16.7 ± 0.6^*∗*^	15.4 ± 0.6^*∗*^	15.1 ± 0.5^*∗*^	14.0 ± 0.5^*∗*^
	7^th^	21.8 ± 0.4	25.8 ± 0.7^*∗*^	22.0 ± 0.4^*∗*^	20.6 ± 0.5^*∗*^	26.2 ± 0.6^*∗*^
	14^th^	8.7 ± 0.3	13.0 ± 0.4^*∗*^	10.0 ± 0.3^*∗*^	9.5 ± 0.2^*∗*^	10.3 ± 0.3^*∗*^

Fibroblast (no./micrograph)	3^rd^	22.5 ± 2.2	39.7 ± 3.0^*∗*^	41.8 ± 1.9^*∗*^	39.2 ± 1.8^*∗*^	38.4 ± 1.7^*∗*^
	7^th^	97.2 ± 2.0	113.0 ± 1.9^*∗*^	115.5 ± 2.9^*∗*^	109.5 ± 1.6^*∗*^	115.8 ± 1.6^*∗*^
	14^th^	66.6 ± 0.6	75.2 ± 0.9^*∗*^	75.3 ± 0.8^*∗*^	77.9 ± 0.9^*∗*^	82.1 ± 1.2^*∗*^

Collagen (%)	3^rd^	17.8 ± 1.1	22.2 ± 1.5	20.9 ± 1.1	19.9 ± 1.8	22.5 ± 2.0
	7^th^	38.1 ± 1.1	42.9 ± 1.8^*∗*^	26.1 ± 1.2^*∗*^	27.6 ± 1.4^*∗*^	39.0 ± 1.3
	14^th^	36.4 ± 1.1	41.9 ± 1.2^*∗*^	44.6 ± 1.3^*∗*^	48.1 ± 1.1^*∗*^	49.0 ± 1.1^*∗*^

Data were analyzed by the MannWhitney test, considering significant values of ^*∗*^*p* < 0.05 when compared with group Tween™ 80 1%.

**Table 6:** Histomorphometric analysis of babassu oil treatment in skin chronic inflammation.

Parameters	Experimental groups			
		Croton oil 5% (20 *µ*L/orelha)		
	Sham (acetone, 20 *µ*L/ear)	Control (acetone, 20 *µ*L/ear)	Babassu oil (10 *µ*L/ear)	Dexametasone (0.1 mg/ear)
*n*	5	9	8	8
Epidermal thickness (µm)	6.0 ± 0.33	48.6 ± 2.44	24.7 ± 1.67^*∗*^	29.4 ± 2.31^*∗*^
Ear thickness (µm)	103.6 ± 5.07	356.3 ± 8.98	217.2 ± 7.00^*∗*^	227.3 ± 13.86^*∗*^

Data expressed as mean ± SEM and analyzed by the MannWhitney test, considering ^*∗*^*p* < 0.05.

## Data Availability

All data used in this study are available from the corresponding author on reasonable request.
